# Photoinactivation of multidrug-resistant Gram-positive bacteria isolated from dogs with otitis using tetra-cationic porphyrins bearing peripheral platinum(II) (**3-PtTPyP**) and palladium(II) (**3-PdTPyP**) complexes

**DOI:** 10.1007/s00284-026-05202-8

**Published:** 2026-11-04

**Authors:** Tauana Fernanda Jantsch Voigt, Eduarda Martins, Junior Navarini, Matheus Gass Lopes, Paulo Ricardo de Souza, Bernardo Almeida Iglesias, Fernanda Silveira Flores Vogel, Letícia Trevisan Gressler, Juliana Felipetto Cargnelutti

**Affiliations:** 1 Center for Rural Sciences, Universidade Federal de Santa Maria, Santa Maria, Rio Grande do Sul Brasil; 2 Laboratory Surfaces and Macromolecules, Department of Physics, Center for Natural and Exact Sciences, Federal University of Santa Maria, Santa Maria, Rio Grande do Sul Brasil; 3 Chemistry Department, Universidade Federal de Santa Catarina, Florianópolis, Santa Catarina Brasil; 4 Department of Preventive Veterinary Medicine, Center for Rural Sciences, Universidade Federal de Santa Maria, Santa Maria, Rio Grande do Sul Brasil; 5 Laboratory of Veterinary Microbiology and Immunology, Federal Institute Farroupilha, Campus Frederico Westphalen, Frederico Westphalen, Rio Grande do Sul Brasil

## Abstract

The increasing prevalence of multidrug-resistant (MDR) bacteria isolated from dogs with external otitis presents a major therapeutic challenge in veterinary medicine, and antimicrobial photodynamic therapy has emerged as a promising alternative. Meso-tetra-pyridyl porphyrins bearing peripheral platinum(II) (**3-PtTPyP**) and palladium(II) (**3-PdTPyP**) complexes have shown activity against clinically relevant Gram-positive bacteria and may represent effective photosensitizers for otitis-associated MDR pathogens. Therefore, this study evaluated the antimicrobial activity and effects on bacterial morphology and adhesive properties of **3-PtTPyP** and **3-PdTPyP** on MDR isolates of *Corynebacterium auriscanis*, *Enterococcus faecalis*, *Staphylococcus epidermidis*, and *Streptococcus canis* obtained from dogs with otitis. Antibacterial activity was investigated by determining the minimum inhibitory and bactericidal concentrations (MBC) and by bacterial time-kill assay under white-light irradiation for 120 min. Alterations in bacterial morphology and nanomechanical properties were investigated using atomic force microscopy (AFM). Both porphyrins promoted a marked reduction in bacterial concentrations under white-light irradiation using MBC, although their bactericidal effects varied depending on the bacterial species and porphyrin. The **3-PtTPyP** exhibited a more consistent photodynamic response against *E. faecalis* e *C. auricanis*, whereas **3-PdTPyP** displayed greater effected against *S. canis* e *S. epidermidis*. AFM revealed pronounced changes in surface topography and nanomechanical properties after treatment with both porphyrins. Overall, these results demonstrate that **3-PtTPyP** and **3-PdTPyP** are promising photosensitizers for the inactivation of MDR Gram-positive bacteria associated with canine otitis externa, and the performance against the MDR Gram-positive bacteria was dependent of species.

## Introduction

The increasing isolation of multidrug-resistant (MDR) bacteria from clinical samples in dogs, particularly in cases of external otitis, represents a significant challenge for veterinary practitioners. This is due not only to the difficulty in identifying effective treatments but also to the potential One Health implications, as resistant bacteria can be transmitted between companion animals and their owners [1–6]. Gram-positive bacteria such as *Corynebacterium auriscanis* (*C. auriscanis*), *Enterococcus faecalis* (*E. faecalis*), *Staphylococcus epidermidis* (*S. epidermidis*), and *Streptococcus canis* (*S. canis*) exhibit high levels of antimicrobial resistance that can compromise conventional therapies and promote chronic or recurrent infections [7–10]. In this context, research into alternative therapeutic strategies capable of targeting MDR bacteria is essential, contributing to improved clinical management and control of these pathogens [11, 12].

Antimicrobial photodynamic therapy (aPDT) has garnered increasing interest as an alternative approach for combating MDR bacteria [13, 14]. This strategy combines a photosensitizer (PS), a light source, and molecular oxygen to generate reactive oxygen species (ROS) that induce oxidative damage to lipids, proteins, and nucleic acids, resulting in microbial inactivation [13, 15]. In addition, since light application is restricted to the affected area, the commensal microbiota in other body regions is largely preserved, unlike what may occur following systemic administration of conventional antimicrobials [16].

Among PSs used in aPDT, tetra-cationic porphyrins have attracted particular interest due to their high affinity for bacterial cells and their ability to efficiently inactivate microorganisms, particularly Gram-positive species [14]. This enhanced susceptibility is commonly attributed to structural features of the Gram-positive cell wall, including a thick peptidoglycan layer and absence of an outer membrane, which favor PS diffusion toward intracellular targets and effective photoinactivation [13]. In contrast, Gram-negative bacteria are less susceptible to photodynamic inactivation due to the presence of an lipopolysaccharide-rich outer membrane, which acts as a permeability barrier and restricts PS uptake [17].

Porphyrins with chemical modifications, especially those incorporating peripheral metal complexes such as platinum(II) and palladium(II), can exhibit enhanced photophysical and photochemical properties, leading to increased ROS production [18–20]. Numerous studies on tetra-cationic porphyrins containing peripheral platinum(II) (**3-PtTPyP**) and palladium(II) (**3-PdTPyP**) complexes have demonstrated their antifungal, antibacterial, and antiviral activities in vitro [21–23]. However, most investigations have largely focused on photoinactivation of human clinical isolates and reference strains and have not included MDR clinical samples from animals. Although the activities of these porphyrins against *Staphylococcus aureus* from humans [22] and *Staphylococcus pseudintermedius* from dogs [24] have been reported, research on other Gram-positive microorganisms associated with canine otitis remains scarce.

Our previous study demonstrated that tetra-cationic porphyrins coordinated with platinum(II) and palladium(II) exhibited greater antimicrobial photodynamic activity against MDR Gram-positive bacteria than against Gram-negative isolates associated with canine otitis. Building on these findings, the present study evaluated the antimicrobial photodynamic activity of the most promising derivatives, of tetra-cationic porphyrins bearing peripheral platinum(II) (**3-PtTPyP**) and palladium(II) (**3-PdTPyP**), against MDR *C. auriscanis*, *E. faecalis*, *S. epidermidis*, and *S. canis* isolated from dogs with otitis. By expanding the range of clinically relevant target microorganisms, this study provides additional evidence of the antimicrobial potential of these porphyrins and further supports their application as promising photosensitizers for the treatment of infections caused by MDR Gram-positive pathogens.

## Materials and Methods

### Experimental Design

Multidrug-resistant (MDR) bacterial isolates from dogs with otitis were subjected to aPDT using two tetra-cationic porphyrins with peripheral platinum(II) (**3-PtTPyP**) and palladium(II) (**3-PdTPyP**) complexes. Antimicrobial activity was evaluated by determining the minimum inhibitory concentration (MIC) and minimum bactericidal concentration (MBC), followed by time-kill assays using the MBC. Additionally, the effects of the porphyrins on bacterial cell morphology and adhesive properties were evaluated by atomic force microscopy (AFM).

### Porphyrin Preparation

**Fig. 1:**
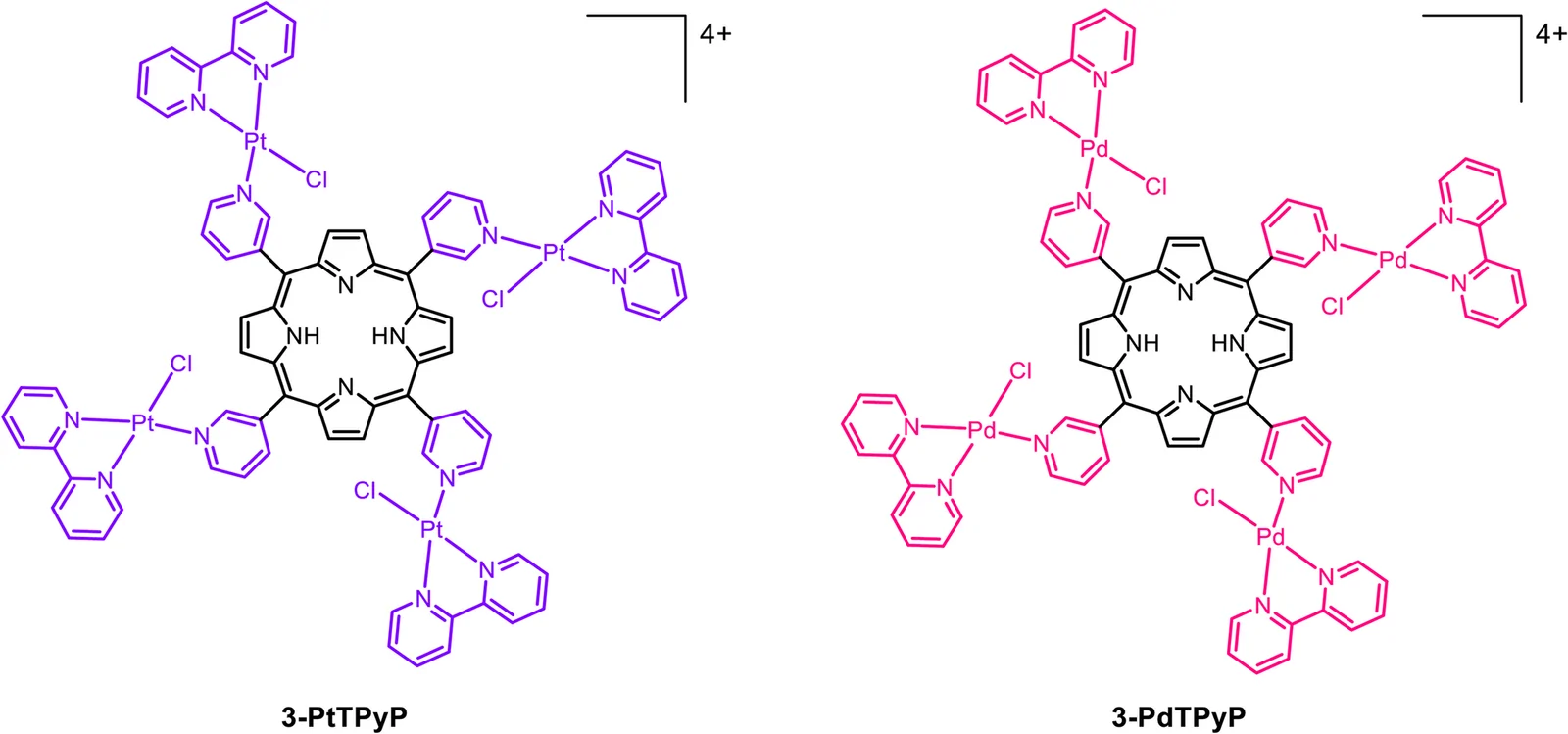
Structural representation of *meta*-substituted *meso*-tetra(pyridyl)porphyrins bearing peripheral platinum(II) and palladium(II) complexes. Counter-ions PF_6_^–^ are omitted for clarity

*Meso*-tetra-pyridyl porphyrins with peripheral platinum(II) (**3-PtTPyP**) and palladium(II) (**3-PdTPyP**) complexes (Fig. 1), both diluted in dimethyl sulfoxide (DMSO), were synthesized and purified as described in the literature [19, 22, 25].

### Irradiation Conditions

All photoirradiation experiments were performed using a custom-built white LED array system (Philips^®^ LEDs; 400–800 nm) at an irradiance of 25 mW/cm², delivering a total light dose of 180 J/cm² over 120 min [19, 22].

### Bacterial Isolate Collection and Characterization

MDR bacterial isolates were recovered from clinical samples obtained from dogs with otitis during routine diagnostic procedures. The study included clinical isolates of *C. auriscanis* (LM 133/22), *E. faecalis* (SBQ 340/23), *S. epidermidis* (SBP 61/24), and *S. canis* (SB 116/18). The isolates were initially identified by phenotypic methods as described by Markey et al. [26], followed by molecular identification based on partial 16 S rRNA gene sequencing [27]. Sequencing was performed using the Sanger method [28], and the resulting sequences were analyzed using the Basic Local Alignment Search Tool (BLAST) to confirm species identity. Antimicrobial susceptibility was determined by the disk diffusion method according to the Clinical and Laboratory Standards Institute (CLSI) [29] and the Brazilian Committee on Antimicrobial Susceptibility Testing (BrCAST) guidelines [30]. Isolates resistant to at least one antimicrobial agent from three or more antimicrobial classes were classified as MDR [31].

### Minimum Inhibitory and Bactericidal Concentrations

MICs were determined using the broth microdilution method, in accordance with CLSI M100-S23 guidelines [32]. Briefly, two-fold serial dilutions of the porphyrins (40–0.019 µM) were prepared in 96-well microplates containing Mueller-Hinton broth for *S. epidermidis* and trypticase soy broth for *C. auriscanis*, *E. faecalis*, and *S. canis*. Subsequently, 50 µL of bacterial suspensions (~ 5 × 10^4^ colony-forming units [CFU]) were added to each well. Positive controls consisted of bacterial inoculum in the corresponding broth, while negative controls contained only broth. A control containing only DMSO was included to rule out any antimicrobial effect of the porphyrin solvent. The plates were kept at room temperature for 120 min of light exposure under the previously mentioned conditions and then incubated under aerobic conditions at 37 °C without agitation. *S. epidermidis*, *S. canis*, and *E. faecalis* were incubated for 24 h, while *C. auriscanis* was incubated for 48 h. To determine the MBC, 10 µL aliquots were collected from each well of the MIC plate and plated onto Mueller-Hinton agar (*S. epidermidis*) or 5.0% sheep blood agar (*C. auriscanis*, *E. faecalis*, and *S. canis*), followed by incubation as previously described. The MBC was defined as the lowest porphyrin concentration at which no detectable bacterial growth was observed after subculturing, whereas the MIC was defined as the lowest concentration that inhibited visible bacterial growth. All assays were performed in triplicate.

### Bacterial Time-Kill Curve

The protocol used for the bacterial time-kill assay was adapted from the methodology described by Seeger et al. [33]. Bacterial time-kill curves were performed to characterize the kinetics of photodynamic bacterial inactivation over 120 min of irradiation, allowing the assessment of both the rate and extent of bacterial killing over time. Porphyrin concentrations used at bacterial time-kill assay corresponding to the previously determined MBCs.

Briefly, 100 µL of bacterial inoculum (~ 5 × 10⁵ CFU/mL, resulting in ~ 5 × 10^4^ CFU/microtube) was mixed with 100 µL of porphyrin solution at the MBC in sterile microtubes. Control samples received 100 µL of the corresponding culture medium instead of the porphyrin solution. Aliquots were collected at 0, 30, 60, 90, and 120 min of white light exposure, with the 0-min baseline samples collected immediately prior to irradiation. Collected aliquots were serially diluted, plated onto the appropriate culture media, and incubated under the aforementioned conditions. Dilution ranges were optimized for each sampling point to yield countable colonies, with a limit of detection of 10^1,301^ CFU/mL per replicate (20 CFU).

### Atomic Force Microscopy Analysis

The effects of porphyrins on bacterial morphology and adhesive properties were analyzed using AFM. Sample preparation followed the procedure described by Fussinger et al. [34]. Porphyrin concentrations were determined for each bacterial isolate based on the previously established MBCs, and half of the MBC (½ MBC) was used for AFM analysis. For each isolate, bacterial suspensions were subjected to the same irradiation conditions described for the time-kill assay. Light control and porphyrin-treated suspensions were both exposed to white light for 120 min, with the latter receiving the porphyrin at ½ MBC. Following irradiation, a 20 µL aliquot of each bacterial suspension was deposited onto freshly cleaved mica substrates (1.0 × 1.0 cm²) for AFM analysis.

The AFM were performed using an NX10 microscope (Park Systems, Korea) with the Smart Scan software (v. 1.0.RTM13c2020-1). Topographic images were acquired in contact mode; adhesion force, deformation, and energy dissipation maps were obtained in PinPoint mode. Topographic and nanomechanical measurements were performed using a high-frequency tip (PPP-NCHR-50, Nanosensors, Bulgaria) with a nominal resonance frequency of 204–497 kHz and a force of 10–130 N m^‒1^. Images were collected from multiple regions of 5.0 × 5.0 µm^2^ and 10.0 × 10.0 µm^2^. Image processing and analyses were conducted offline using the Gwyddion software (v. 2.70). The average root mean square roughness (Rq), deformation, adhesion force, and adhesion energy values were calculated from at least six non-overlapping regions per sample. All measurements were performed at room temperature (25 ± 5 °C) and relative humidity (50 ± 5.0%).

### Statistical Analysis

The effects of treatment and irradiation time on bacterial time-kill assays were analyzed by two-way analysis of variance (two-way ANOVA), followed by Tukey’s multiple comparisons test, using GraphPad Prism version 6.0. Differences were considered statistically significant when *p* < 0.05. All assays were performed in triplicate.

## Results

The antimicrobial susceptibility profiles of the clinical isolates are presented in Table 1. Molecular identification based on partial 16 S rRNA gene sequencing revealed high similarity with sequences deposited in GenBank. *C. auriscanis* (LM 133/22) showed 100% identity with the complete genome sequence of *C. auriscanis* reference genome (CP047046.1); the *E. faecalis* isolate (SBQ 340/23) also exhibited 100% identity with the complete *E. faecalis* genome (CP174869.1); *S. epidermidis* (SBP 61/24) showed 99.75% identity with the complete *S. epidermidis* genome (CP097519.1); and *S. canis* (SB 116/18) showed 100% identity with the corresponding reference genome (CP053792.1). These results confirm the accurate molecular identification of the bacterial isolates.

**Table 1:** Antimicrobial susceptibility profiles of the clinical isolates included in the antimicrobial activity assays with the porphyrins 3-PtTPyP and 3-PdTPyP

Bacteria	Susceptibility profile to antimicrobial agents		
	Sensitive	Intermediate	Resistant
*C. auriscanis*	RIF^b^, TET^b^		CFO^b^, CIP^b^, CLI^b^, ERI^b^, MER^b^, SUT^b^
*E. faecalis*	CLO^a^, LEV^b^, NOR^b^	CIP^b^	DOX^a^, IPM^b^, PEN^a^, TET^a^
*S. epidermidis*	CIP^b^, CLI^a^, CLO^a^, GEN^b^, LEV^b^, NEO^a^, TOB^b^		AZI^a^, BA^a^, CFO^a^, CLA^a^, DOX^a^, ERI^b^, OXA^a^, PEN^a^, POL^a^, SUT^b^, TET^b^
*S. canis*	ERI^a^, CVN^a^	CLI^a^, ENO^a^, MRB^a^	DOX^a^, ORB^a^, RIF^b^, TET^a^

^a^Antimicrobial susceptibility testing interpretive criteria established by CLSI (2024) [29]. ^b^Antimicrobial susceptibility testing interpretive criteria defined by BrCAST (2025) [30]. Antimicrobials tested: amikacin, 30 µg (AMI); azithromycin, 15 µg (AZI); bacitracin, 10 µg (BA); cephalexin, 30 µg (CFE); cefotaxime, 30 µg (CFO); cefovecin, 30 µg (CVN); ciprofloxacin, 5.0 µg (CIP); clarithromycin, 15 µg (CLA); clindamycin, 2.0 µg (CLI); chloramphenicol, 30 µg (CLO); doxycycline, 30 µg (DOX); enrofloxacin, 5.0 µg (ENO); erythromycin, 15 µg (ERI); gentamicin, 10 µg (GEN); imipenem, 10 µg (IPM); levofloxacin, 5.0 µg (LEV); meropenem, 10 µg (MEM); neomycin, 30 µg (NEO); oxacillin, 1.0 µg (OXA); orbifloxacin, 10 µg (ORB); penicillin, 10 IU (PEN); polymyxin B, 300 IU (POL); rifampicin, 5.0 µg (RIF); trimethoprim-sulfamethoxazole, 25 µg (SUT); tetracycline, 30 µg (TET); tobramycin, 10 µg (TOB); vancomycin, 30 µg (VAN)

The MICs and MBCs of **3-PtTPyP** and **3-PdTPyP** against the clinical isolates, under both dark conditions and white light irradiation, are presented in Table 2. Under dark conditions, no detectable antimicrobial effect was observed at the concentrations tested for any of the isolates (not shown). Under irradiation, **3-PtTPyP** exhibited lower MBC values than **3-PdTPyP** against *E. faecalis* (SBQ 340/23), *S. epidermidis* (SBP 61/24), and *S. canis* (SB 116/18). In contrast, for *C. auriscanis* (LM 133/22), both porphyrins exhibited identical MBC values under irradiation (Table 2).

**Table 2:** Minimum inhibitory (MIC) and bactericidal concentrations (MBC) of **3-PtTPyP** and **3-PdTPyP **against clinical isolates under irradiation^a^

Bacteria	3-PtTPyP		3-PdTPyP	
	MIC (µM)	MBC (µM)	MIC (µM)	MBC (µM)
*C. auriscanis* (LM 133/22)	0.625	0.625	0.625	0.625
*E. faecalis* (SBQ 340/23)	1.25	1.25	2.5	2.5
*S. epidermidis* (SBP 61/24)	2.5	2.5	20	20
*S. canis* (SB 116/18)	0.625	0.625	1.25	1.25

*MIC* minimum inhibitory concentrations, *MBC* minimal bactericidal concentration

^a^Irradiation was performed using a custom-built white light LED array system (Philips^®^ LEDs; 400–800 nm) at 25 mW/cm^2^, delivering a total light dose of 180 J/cm^2^ over 120 min

**Fig. 2:**
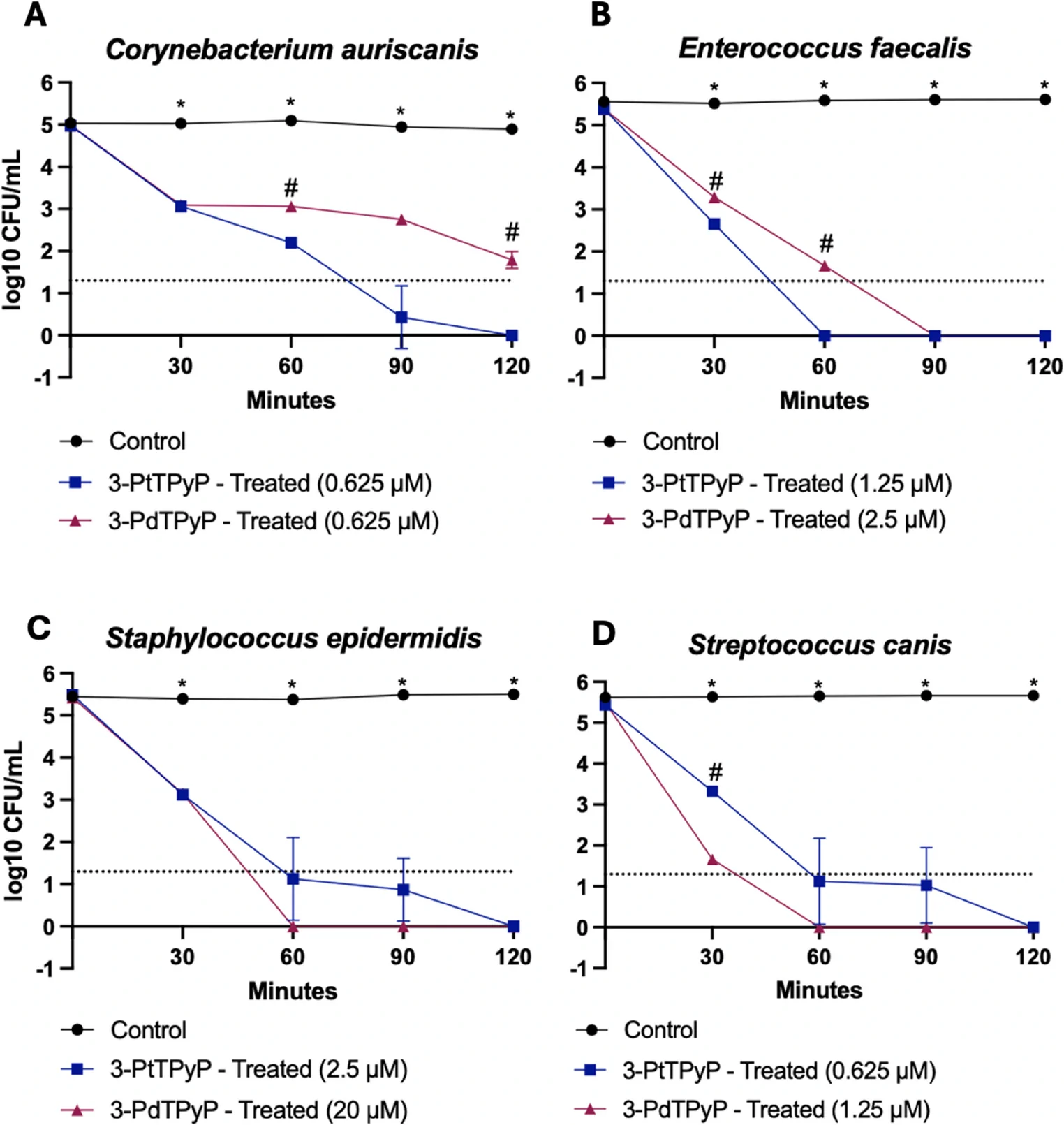
Bacterial time-kill curves of multidrug-resistant isolates following photodynamic treatment with **3-PtTPyP** and** 3-PdTPyP** under white-light irradiation (25 mW/cm²; 180 J/cm²). **A***C. auriscanis* (LM 133/22) treated with **3-PtTPyP **(0.625 µM) and **3-PdTPyP** (0.625 µM); **B***E. faecalis* (SBQ 340/23) treated with **3-PtTPyP** (1.25 µM) and **3-PdTPyP** (2.5 µM); **C***S. epidermidis* (SBP 61/24) treated with **3-PtTPyP** (2.5 µM) and **3-PdTPyP** (20 µM); and **D***S. canis* (SB 116/18) treated with **3-PtTPyP** (0.625 µM) and **3-PdTPyP** (1.25 µM). Data are expressed as log₁₀ CFU/mL, with black circles representing light controls (non-porphyrin treated), blue squares representing **3-PtTPyP**-treated groups, and pink triangles representing **3-PdTPyP**-treated groups. The light controls were exposed to the same irradiation conditions as the treated groups. *Indicate significant differences between the control and treated groups. #Indicate significant differences between porphyrin treatments (*p* < 0.05). Grid line indicates the assay limit of detection (10^1.301^ CFU/mL) per replicate. Bars indicate standard deviation (SD) of the mean

In the bacterial time-kill assay, the light-exposed control groups maintained stable bacterial concentrations throughout the irradiation period, whereas treatment with both porphyrins at their respective MBCs resulted in pronounced antimicrobial activity against all MDR isolates (Fig. 2).

For *C. auriscanis* (LM 133/22), treatment with **3-PtTPyP** at the MBC (0.625 µM) resulted in a progressive reduction in bacterial viability, with no detectable bacterial growth observed after 120 min of irradiation (Fig. 2A). Although **3-PdTPyP **at its MBC (0.625 µM) also reduced bacterial viability, its photodynamic effect was slower, with bacterial viability remaining detectable after 120 min of irradiation (Fig. 2A).

In the *E. faecalis* time-kill curves, the treatment with **3-PtTPyP** at MBC (1.25 µM) resulted in a rapid reduction in bacterial viability, with no detectable bacterial growth observed after 60 min of irradiation (Fig. 2B). Similarly, **3-PdTPyP **also reduced bacterial viability at the MBC (2.5 µM); however, no detectable bacterial growth was observed only after 90 min of irradiation (Fig. 2B).

For *S. epidermidis*, treatment with **3-PtTPyP** at its MBC (2.5 µM) resulted in a marked reduction in bacterial viability, with no detectable bacterial growth after 120 min of irradiation (Fig. 2C). In contrast, **3-PdTPyP** (20 µM) exhibited faster antimicrobial activity, with no detectable bacterial growth after 60 min of irradiation (Fig. 2C).

In the bacterial time-kill curve of *S. canis*, treatment with **3-PtTPyP** (0.625 µM) resulted in a rapid reduction in bacterial viability after 30 min of irradiation, with no detectable bacterial growth after 120 min (Fig. 2D). Similarly, treatment with **3-PdTPyP** (1.25 µM) also reduced bacterial viability, with no detectable bacterial growth after 60 min of irradiation (Fig. 2D).

The effects of **3-PtTPyP** and **3-PdTPyP** were evaluated at ½ MBC against *C. auriscanis*, *E. faecalis*, *S. epidermidis*, and *S. canis*. Bacterial morphological and nanomechanical properties were assessed by AFM, including root mean square roughness (Rq), deformation, adhesion force, and adhesion energy, in both untreated control and porphyrin-treated groups following light exposure. Standard deviations are based on six measurements (Tables 3, 4, 5 and 6).

**Table 3:** Atomic force microscopy parameters of *C. auriscanis* before and after treatment with **3-PtTPyP **and **3-PdTPyP** under irradiation

Bacteria	Rq (nm)	Deformation (nm)	Adhesion force (nN)	Adhesion energy (fJ)
Control	51.16 ± 8.66	14.60 ± 0.06	72.83 ± 2.31	0.43 ± 0.01
**3-PtTPyP**	20.58 ± 3.72	4.49 ± 0.42	461.42 ± 14.02	5.74 ± 0.26
**3-PdTPyP**	3.30 ± 0.12	0.64 ± 0.16	834.75 ± 66.88	11.46 ± 1.58

**Table 4:** Atomic force microscopy parameters of *E. faecalis* before and after treatment with **3-PtTPyP** and **3-PdTPyP** under irradiation

Bacteria	Rq (nm)	Deformation (nm)	Adhesion force (nN)	Adhesion energy (fJ)
Control	12.25 ± 0.74	6.98 ± 1.16	268.00 ± 7.33	2.70 ± 0.34
**3-PtTPyP**	10.54 ± 2.16	5.25 ± 0.48	222.63 ± 25.44	3.10 ± 0.08
**3-PdTPyP**	8.84 ± 0.71	2.90 ± 0.06	310.16 ± 9.64	3.35 ± 0.28

**Table 5:** Atomic force microscopy parameters of *S. epidermidis* before and after treatment with **3-PtTPyP** and **3-PdTPyP** under irradiation

Bacteria	Rq (nm)	Deformation (nm)	Adhesion force (nN)	Adhesion energy (fJ)
Control	35.53 ± 4.95	25.75 ± 6.72	73.50 ± 21.82	0.52 ± 0.21
**3-PtTPyP**	23.25 ± 3.77	12.01 ± 1.25	284.97 ± 38.35	4.13 ± 0.10
**3-PdTPyP**	22.80 ± 1.83	3.96 ± 0.60	668.85 ± 27.01	9.27 ± 2.40

**Table 6:** Atomic force microscopy parameters of *S. canis* before and after treatment with **3-PtTPyP** and **3-PdTPyP** under irradiation

Bacteria	Rq (nm)	Deformation (nm)	Adhesion force (nN)	Adhesion energy (fJ)
Control	63.23 ± 6.62	3.54 ± 0.25	268.66 ± 10.65	2.25 ± 0.13
**3-PtTPyP**	13.15 ± 1.27	4.93 ± 0.75	1645.83 ± 88.78	49.44 ± 4.64
**3-PdTPyP**	13.72 ± 2.81	7.16 ± 1.06	185.13 ± 7.08	1.49 ± 0.17

**Fig. 3:**
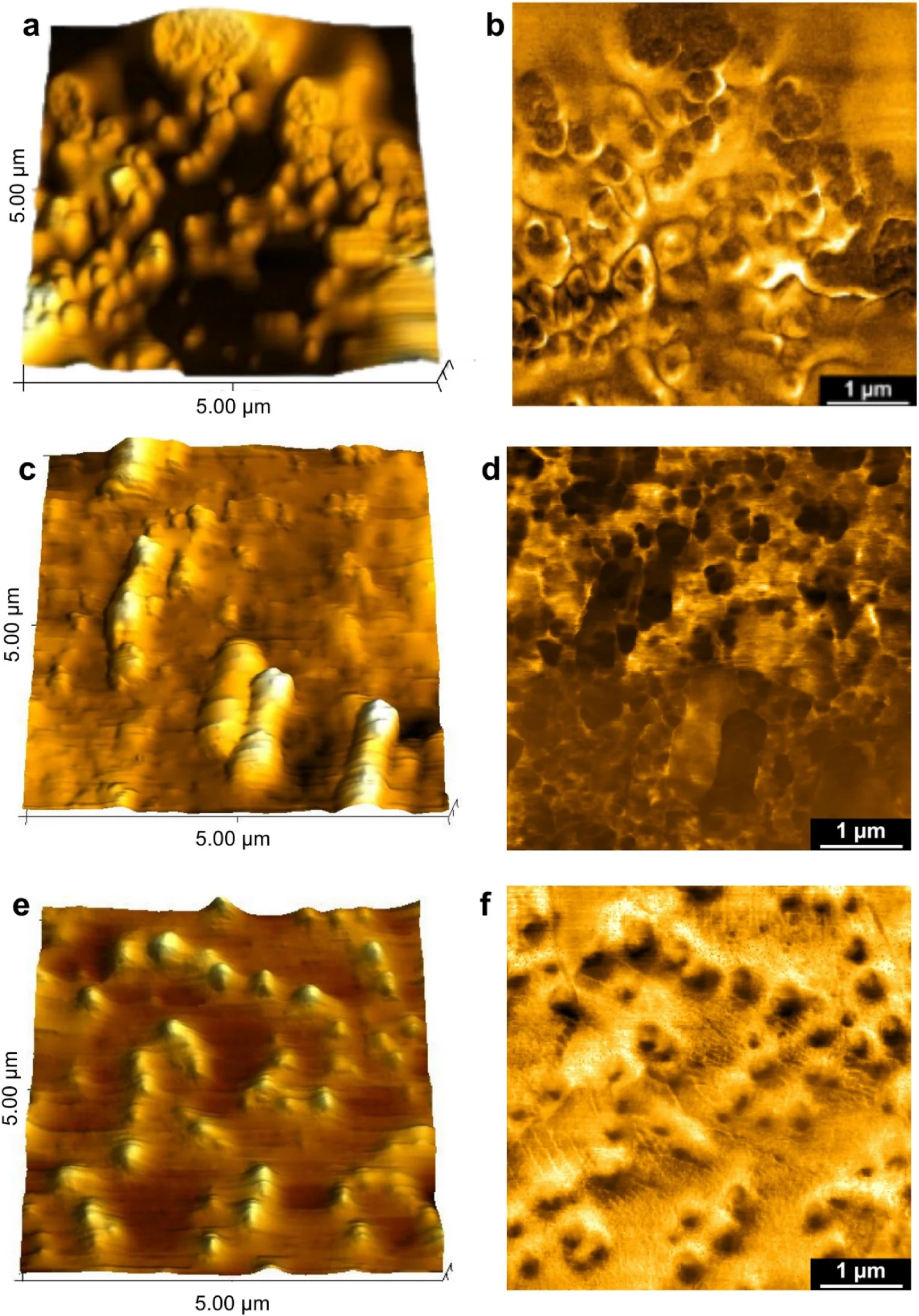
Representative AFM images of *C. auriscanis* after the different experimental treatments. The left panels (**a**, **c**, and **e**) show surface topography, while the right panels (**b**, **d**, and **f**) show the corresponding deformation maps. All images were acquired over a 5 × 5 μm scan area; scale bar = 1.0 μm. **a**, **b** untreated control; **c**, **d** cells treated with **3-PtTPyP** under light irradiation; and **e**, **f** cells treated with **3-PdTPyP** under light irradiation

**Fig. 4:**
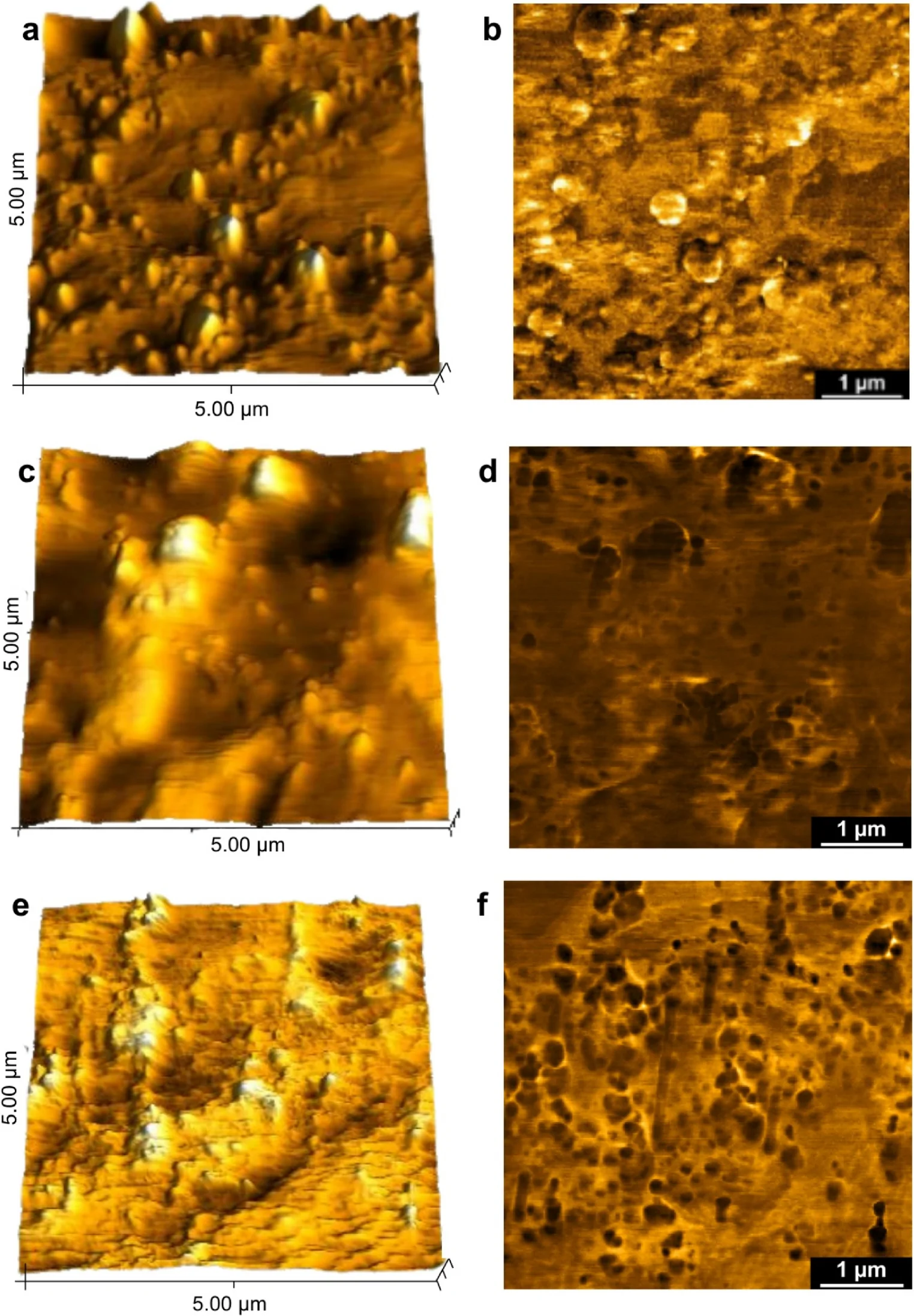
Representative AFM images of *E. faecalis* after the different experimental treatments. The left panels (**a**, **c**, and **e**) show surface topography, while the right panels (**b**, **d**, and **f**) show the corresponding deformation maps. All images were acquired over a 5 × 5 μm scan area; scale bar = 1.0 μm. **a**, **b** untreated control; **c**, **d** cells treated with **3-PtTPyP** under light irradiation; and **e**, **f** cells treated with **3-PdTPyP** under light irradiation

**Fig. 5:**
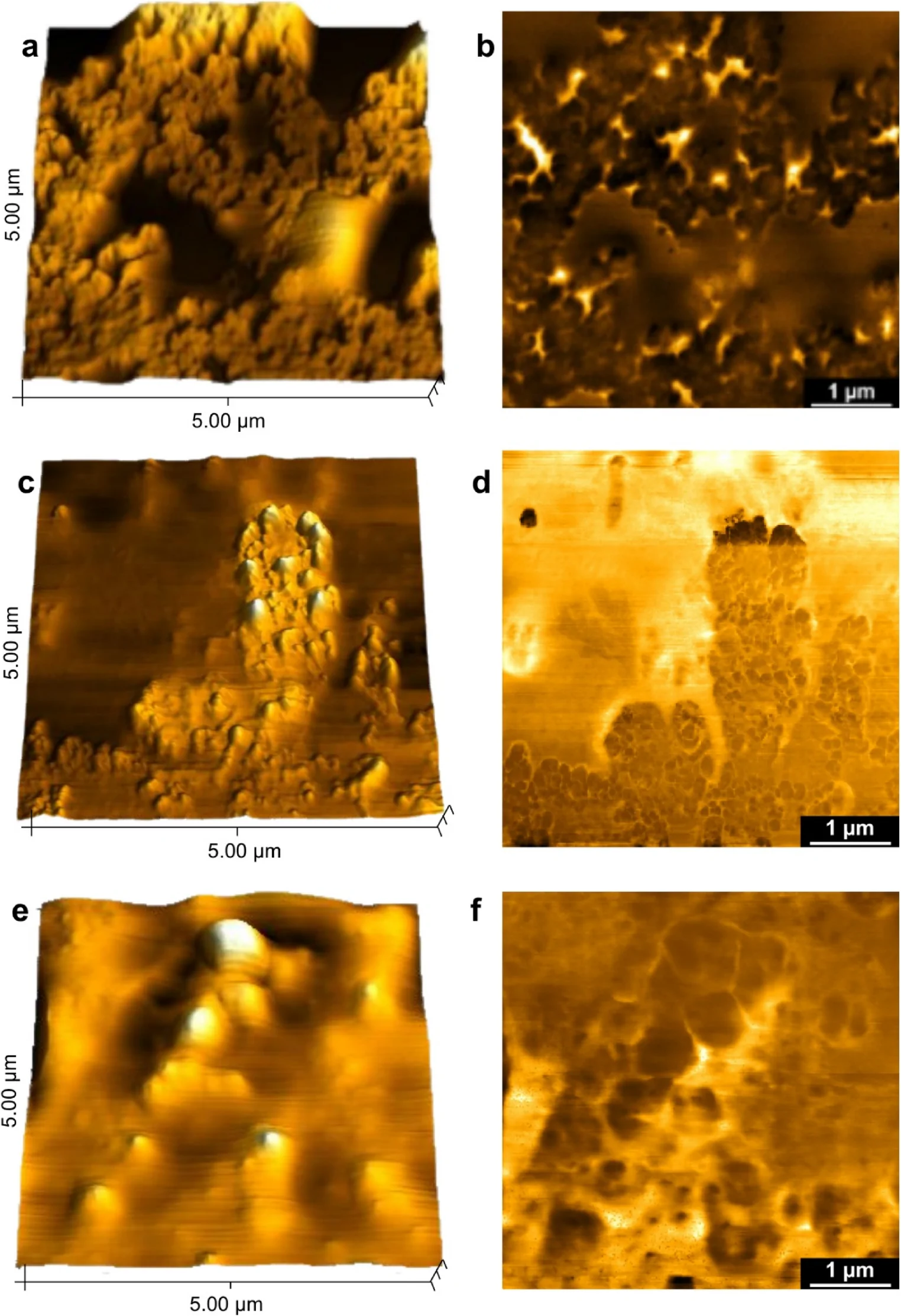
Representative AFM images of *S. epidermidis* after the different experimental treatments. The left panels (**a**, **c** and **e**) show surface topography, while the right panels (**b**, **d**, and **f**) show the corresponding deformation maps. All images were acquired over a 5 × 5 μm scan area; scale bar = 1.0 μm. **a**, **b** untreated control; **c**, **d** cells treated with **3-PtTPyP** under light irradiation; and **e**, **f** cells treated with **3-PdTPyP** under light irradiation

**Fig. 6:**
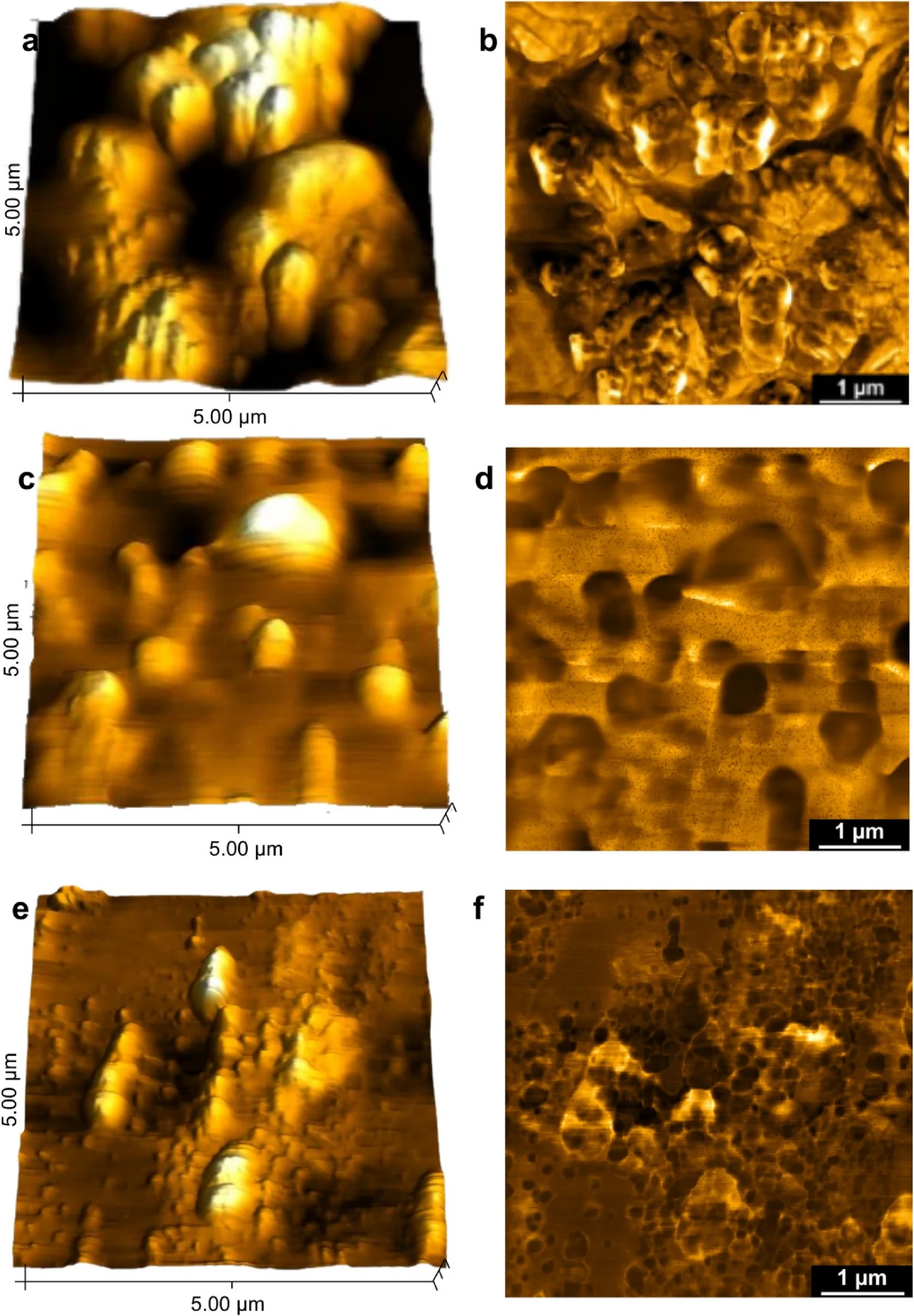
Representative AFM images of *S. canis* after the different experimental treatments. The left panels (**a**, **c** and **e**) show surface topography, while the right panels (**b**, **d** and **f**) show the corresponding deformation maps. All images were acquired over a 5 × 5 μm scan area; scale bar = 1.0 μm. **a**, **b** untreated control; **c**, **d** cells treated with **3-PtTPyP** under light irradiation; and **e**, **f** cells treated with **3-PdTPyP **under light irradiation

The Rq roughness values varied with the clinical isolate and type of porphyrin used. For *C. auriscanis*, a marked decrease in surface roughness was observed following treatment with **3-PtTPyP** under light exposure, with Rq values decreasing from 51.16 nm (control) to 20.58 nm, and a higher Rq value decrease to 3.30 nm for **3-PdTPyP **treatment under light exposure (Table 3; Fig. 3). In *E. faecalis*, roughness decreased after treatment with **3-PtTPyP** and **3-PdTPyP **under light conditions, from 12.25 nm (control) to 10.54 and 8.84 nm, respectively (Table 4; Fig. 4). For *S. epidermidis* (Table 5; Fig. 5), roughness was reduced following exposure to both **3-PtTPyP** and **3-PdTPyP** under light conditions, with Rq values of 23.25 and 22.80 nm, respectively, compared to 35.53 nm for the control. For *S. canis* (Table 6; Fig. 6), a significant reduction in Rq was observed after treatment with both **3-PtTPyP **and **3-PdTPyP** under light irradiation, with Rq values ranging from 63.23 nm (control) to 13.15 nm (**3-PtTPyP**) and 13.72 nm (**3-PdTPyP**).

Analysis of cell deformation revealed significant changes after light conditions, indicating altered mechanical responses of the bacteria to AFM tip-applied force. Overall, the deformation results indicate that treatment with porphyrins induces substantial modifications in bacterial mechanical properties, which vary according to the MDR clinical isolate, porphyrin type, and light irradiation. Under light exposure, treatment with **3-PdTPyP **resulted in decreased deformation values for *C. auriscanis*, *E. faecalis*, and *S. epidermidis*: from 14.60 nm (control) to 0.64 nm for *C. auriscanis*, from 6.98 nm (control) to 2.90 nm for *E. faecalis*, and from 25.75 nm (control) to 3.96 nm for *S. epidermidis*.

Adhesion force and adhesion energy were also markedly affected by porphyrin treatment, with variations depending on the bacterial isolate and porphyrin type. For *C. auriscanis* (Table 3), both adhesion force and energy increased after treatment with **3-PtTPyP** and **3-PdTPyP** under light: from 72.83 nN and 0.43 fJ (control) to 461.42 nN and 5.74 fJ following **3-PtTPyP** treatment, and to 834.75 nN and 11.46 fJ after treatment with **3-PdTPyP**. For *E. faecalis* (Table 4), a reduction in adhesion force and an increase in adhesion energy were observed following treatment with **3-PtTPyP** under light compared with untreated bacteria. Control cells exhibited adhesion force and energy values of 268.00 nN and 2.70 fJ, whereas treated cells showed 222.63 nN and 3.10 fJ, respectively. For *S. epidermidis* (Table 5), both parameters increased after exposure to either porphyrin compared to the control. In *S. canis* (Table 6), untreated bacteria exhibited an adhesion force and energy of 268.66 nN and 2.25 fJ, respectively. In contrast, treatment with **3-PtTPyP** resulted in marked increases, with adhesion parameters reaching 1645.83 nN and 49.44 fJ for adhesion force and energy, respectively.

## Discussion

Gram-positive bacteria, including *Corynebacterium* spp., *Enterococcus* spp., *Staphylococcus* spp., and *Streptococcus* spp., are frequently associated with canine otitis externa and may contribute to the persistence and recurrence of infection [6]. The emergence of antimicrobial-resistant and multidrug-resistant (MDR) strains further complicates the management of canine otitis by reducing the effectiveness of conventional antimicrobial therapy and limiting therapeutic options [35, 36]. Therefore, the marked reduction or inactivation of the MDR isolates evaluated in the present study following treatment with **3-PtTPyP** and **3-PdTPyP** is particularly relevant. These findings demonstrate that both porphyrins can effectively target MDR Gram-positive bacteria associated with canine otitis, supporting their potential as alternative or complementary approaches for the management of infections that may be difficult to control with conventional antimicrobials.

Our findings demonstrated that both tested porphyrins (**3-PtTPyP** and **3-PdTPyP**) exhibited promising photodynamic activity against the Gram-positive bacteria *C. auriscanis*, *E. faecalis*, *S. epidermidis*, and *S. canis*. These findings are consistent with the literature, as it is well documented that aPDT using tetra-cationic porphyrins is generally more effective against Gram-positive than Gram-negative bacteria [19, 22, 33, 37, 38]. This differential susceptibility has been associated with differences in cell envelope structure, particularly the absence of an outer membrane and the relatively porous peptidoglycan layer of Gram-positive bacteria, which may facilitate photosensitizer (PS) access to the bacterial surface [13]. As a result, PS molecules can reach the bacterial surface and generate ROS, particularly singlet oxygen (^1^O_2_), in close proximity to key cellular targets such as the membrane, cell wall, and cytoplasm, the localized ROS production induces oxidative damage to these structures, resulting in bacterial inactivation [39]. Similar photoinactivation of Gram-positive bacteria isolated from canine otitis has previously been demonstrated using tetra-cationic porphyrins, supporting the potential of this approach against MDR isolates [24, 33].

Although the experiments were conducted for a total of 120 min, *S. canis*, *S. epidermidis*, and *E. faecalis* were inactivated to the detection limit within 60 min, depending on the porphyrin used, whereas *C. auriscanis* required 120 min to reach the detection limit. This difference in photoinactivation kinetics may be related to species-specific characteristics of the bacterial cell envelope, as the composition and organization of external structures can influence the interaction of the photosensitizer with the bacterial cell and, consequently, its susceptibility to ROS [40]. Considering that **3-PtTPyP** showed greater efficacy against *C. auriscanis* and *E. faecalis*, whereas **3-PdTPyP** was more effective against *S. epidermidis* and *S. canis*, the complementary activity observed between the two porphyrins suggests that their combination may represent an interesting strategy to broaden the photoinactivation spectrum against different MDR bacteria associated with canine otitis.

Previous studies have demonstrated that combining different photosensitizers can enhance bacterial inactivation compared with the use of individual compounds, although the effect of such combinations may vary depending on the microorganism and experimental conditions [41]. In this context, future studies using checkerboard assays could determine whether the combination of **3-PtTPyP** and **3-PdTPyP** produces a synergistic or antagonistic effect, as well as identify the most effective concentrations for the inactivation of different MDR isolates.

A greater efficacy of **3-PtTPyP** was observed against *C. auriscanis* and *E. faecalis*, whereas **3-PdTPyP** exhibited greater activity against *S. epidermidis* and *S. canis*. Although previous studies have reported **3-PtTPyP **as a highly effective photosensitizer against Gram-positive bacteria, including *Enterococcus* spp. and *Staphylococcus* spp. [19], the present findings indicate that this superiority is not uniform across bacterial isolates. This suggests that the photodynamic response may depend not only on the intrinsic properties of the photosensitizer but also on its interaction with specific characteristics of each bacterial isolate [42].

Incorporation of platinum(II) into the porphyrin ring can significantly influence the photochemical and photophysical properties of these molecules [40]. In general, pyridyl-porphyrins lacking peripheral complexes exhibit an increased triplet-state quantum yield. Conversely, porphyrin derivatives containing peripheral Pd(II) or Pt(II) complexes show a reduction in triplet-state quantum yield—particularly in *para* substituted derivatives—with internal conversion serving as the primary deactivation mechanism for the first excited state. Enhanced internal conversion in these porphyrins can extend effective electronic delocalization, thereby favoring non-radiative decay rates and suppressing the radiative pathway [36]. These complexes generally exhibit reduced aggregation, greater photostability under irradiation, and improved solubility (phenomenon dependent on the isomeric position), all of which facilitate PS transport and cellular uptake internalization [21].

Although aPDT shows potential as a therapeutic option for MDR bacteria in cases of external otitis, its clinical translation is influenced by factors that also affect conventional antimicrobial therapies. aPDT efficacy depends directly on adequate PS delivery and effective light penetration into the target tissue [13]. Nonetheless, factors such as the variable anatomy of the canine ear canal, the presence of exudate, and intense inflammation may compromise these aspects, hindering PS diffusion and ROS generation [18]. Additionally, the need for specific equipment, lack of standardized protocols for light dose and PS concentrations, and the time required for animal restraint or sedation represent practical challenges to the routine clinical use of aPDT in veterinary clinical practice, reinforcing the need for further in vivo studies [37].

Complementary to the microbiological findings, the AFM results indicate that treatment with **3-PtTPyP** and **3-PdTPyP** promotes changes in surface topography and bacterial nanomechanical properties following light irradiation. These effects likely reflect electrostatic interactions between the cationic porphyrins and the negatively charged bacterial cell envelope [42, 43]. These interactions, combined with light irradiation, become even more pronounced effects, with damage to the cells’ superficies elements although they do not exhibit a uniform pattern among the different MDR clinical isolates, possibly due to structural particularities of each microorganism’s cell surface.

Contact with porphyrins may reduce electrostatic repulsion among anionic cell wall components (e.g., teichoic acids and peptidoglycan), thereby favoring surface reorganization of the extracellular membrane and decreasing cell deformability [13, 44]. The observed changes in Rq values further support this surface reorganization, reflected in reduced membrane roughness. The absence of a consistent pattern in adhesion strength and energy values after light irradiation suggests the coexistence of photodynamic and non-photodynamic mechanisms at subinhibitory concentrations, including electrostatic interactions and membrane insertion of the porphyrins [40]. Furthermore, light activation may induce both oxidative damage and adaptive cellular responses, enabling identification of intermediate states of bacterial damage. Together, these findings indicate that porphyrin-based treatments alter multiple cellular parameters and promote the reorganization of bacterial surface adhesive components, such as proteins, peptidoglycan, and polysaccharides [35].

## Conclusion

Our study demonstrated that using tetra-cationic porphyrins bearing peripheral platinum(II) (**3-PtTPyP**) and palladium(II) (**3-PdTPyP**) complexes are promising photosensitizers for the photoinactivation of MDR Gram-positive bacteria isolated from canine external otitis. Both porphyrins promoted a marked reduction in bacterial concentrations under white-light irradiation, although their bactericidal effects varied depending on the bacterial species and porphyrin. AFM analysis further demonstrated that both porphyrins induced significant changes in the surface topography and nanomechanical properties of the clinical isolates. From a clinical perspective, antimicrobial photodynamic therapy represents a promising adjunctive treatment for canine otitis caused by MDR bacteria. However, further in vivo studies, and investigations involving biofilms and polymicrobial infections, are needed to validate its therapeutic efficacy and optimize treatment protocols for routine veterinary practice.

## Data Availability

No datasets were generated or analysed during the current study.
